# Epigenetic Bases of Aberrant Glycosylation in Cancer

**DOI:** 10.3390/ijms18050998

**Published:** 2017-05-06

**Authors:** Fabio Dall’Olio, Marco Trinchera

**Affiliations:** 1 Department of Experimental, Diagnostic and Specialty Medicine (DIMES), University of Bologna, 40126 Bologna, Italy; 2 Department of Medicine and Surgery (DMC), University of Insubria, 21100 Varese, Italy

**Keywords:** glycome, glycosyltransferases, DNA methylation, miRNA targeting

## Abstract

In this review, the sugar portions of glycoproteins, glycolipids, and glycosaminoglycans constitute the glycome, and the genes involved in their biosynthesis, degradation, transport and recognition are referred to as “glycogenes“. The extreme complexity of the glycome requires the regulatory layer to be provided by the epigenetic mechanisms. Almost all types of cancers present glycosylation aberrations, giving rise to phenotypic changes and to the expression of tumor markers. In this review, we discuss how cancer-associated alterations of promoter methylation, histone methylation/acetylation, and miRNAs determine glycomic changes associated with the malignant phenotype. Usually, increased promoter methylation and miRNA expression induce glycogene silencing. However, treatment with demethylating agents sometimes results in silencing, rather than in a reactivation of glycogenes, suggesting the involvement of distant methylation-dependent regulatory elements. From a therapeutic perspective aimed at the normalization of the malignant glycome, it appears that miRNA targeting of cancer-deranged glycogenes can be a more specific and promising approach than the use of drugs, which broad target methylation/acetylation. A very specific type of glycosylation, the addition of GlcNAc to serine or threonine (O-GlcNAc), is not only regulated by epigenetic mechanisms, but is an epigenetic modifier of histones and transcription factors. Thus, glycosylation is both under the control of epigenetic mechanisms and is an integral part of the epigenetic code.

## 1. Introduction

As a part of the genome, genes involved in the biosynthesis, degradation, transport, and recognition of the sugar portions of glycoconjugates (collectively referred to as glycogenes) undergo epigenetic regulation. While the simple gene expression/protein expression model could at best account for primary gene products, the existence of epigenetic mechanisms adds another layer of complexity and opportunity for regulating biological functions. Since glycans are not primary gene products, but secondary products of a complex interplay among glycogenes, the epigenetic regulation of the glycome moves a step forward to understanding the complexity and opportunity of biological regulation. It has been hypothesized that the epigenetic regulation of glycogenes provides mammalians with a quick and heritable mechanism generating glycome plasticity to cope with microorganisms [1,2]. As a consequence of epigenetic regulation of glycogenes in solid tissues, the whole glycome of plasma proteins can be modulated. This leads to important functional changes, as well as to the appearance of disease-related glycomic markers [3,4,5]. In light of the enormous impact of epigenetics on human cancers, which are very frequently associated with dramatic deregulation of glycan expression, a growing number of reports have focused on the epigenetic mechanisms affecting glycosylation in cancer.

In this review, we discuss the glycogenes that were found to be regulated by promoter methylation [6,7,8], histone methylation/acetylation [9,10,11], and microRNAs [12,13], in cancer. We see how different epigenetic mechanisms converge to regulate specific pathways of cancer-associated carbohydrate structures. We also summarize the case of O-linked GlcNAc, in which glycosylation is not only the target of epigenetic mechanisms, but contributes to the generation of the epigenetic code. We expect that the relatively small number of glycogenes epigenetically modulated in cancer so far identified are only a minority of those actually undergoing epigenetic regulation.

## 2. Epigenetic-Regulation of Cancer Associated Structures

### 2.1. Core Glycosylation of N-Linked Chains

In this section, we will discuss the epigenetic regulation of three well-known cancer associated carbohydrate structures present in the internal core of N-glycans.

#### 2.1.1. Core Fucosylation

**Figure 1:**
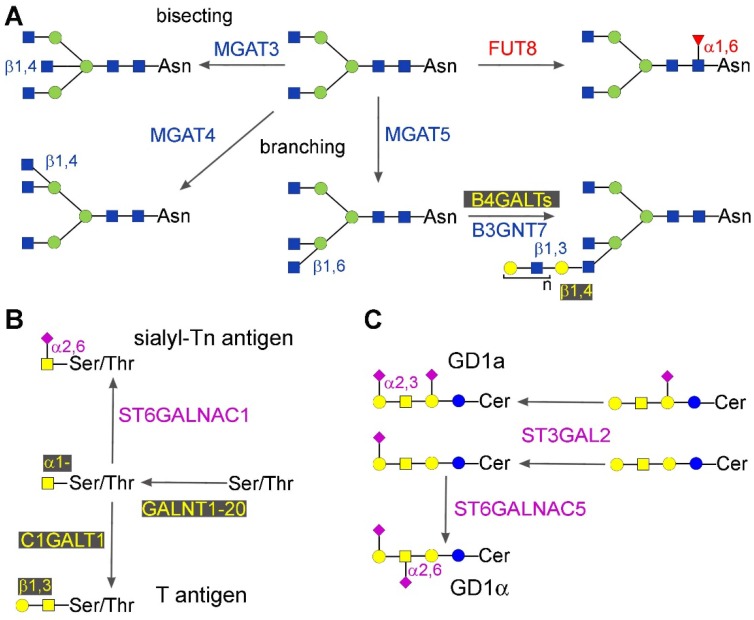
Structure of some core glycans. Monosaccharides are depicted according to this representation: blue square—GlcNAc, N-acetylglucosamine; yellow square—GalNAc, N-acetylgalactosamine; yellow circle—Gal, galactose; blue circle—Glc, glucose; green circle—Man, mannose; red triangle—Fuc, fucose; pink diamond—sialic acid, Sia. Anomers, linkage positions, and enzymes involved in relevant reactions are indicated. (**A**) N-glycans. As an example, the reactions are indicated using only the simple bi-antennary core structure as the substrate. Note that they are not alternative and can occur in various orders, because many of the indicated products can act as the substrate for several of the reported enzymes. An exception is represented by MGAT3 (bisecting enzyme) and MGAT5 (branching enzyme), whose reactions are mutually exclusive; (**B**) O-glycans; (**C**) Gangliosides. In both panels, the enzymes are indicated in the order in which they act.

Core fucosylation consists in the addition of a Fuc residue α1,6-linked to the innermost GlcNAc residue of N-linked chain (Figure 1A). The biosynthesis of this structure is due to a single enzyme: fucosyltransferase 8, encoded by the *FUT8* gene [14]. In mice, the genetic ablation of *FUT8* dysregulates TGF-β signaling, leading to abnormal lung development and emphysema-like phenotype [15]. Increased core fucosylation has been reported during hepatocarcinogenesis, in both cell-associated and secreted proteins, including α-fetoprotein and α1-antitrypsin, which are plasma markers of the disease [16]. However, the expression of *FUT8* in cancer biology is double-hedged—it can be associated with both increased [17] or decreased [18] malignancy in different tissues. *FUT8* is the target of different miRNA. In colorectal cancer, miR-198 up-regulates *FUT8* expression at both the mRNA and protein level, leading to an invasive phenotype [19]. In liver cancer cells, *FUT8* appears to be down-regulated by miR-122, miR-34a [20], miR-26a, miR-34a, and miR-146a [21]. In some cases, miR-34a modifies protein but not mRNA levels [20], showing that some miRNAs can exert their effects only at a translational level.

#### 2.1.2. Bisecting GlcNAc, β1,6 and β1,4 Branching

Bisecting GlcNAc consists of a GlcNAc residue β1,4-linked to the innermost Man residue of the N-glycan core (Figure 1A). Its biosynthesis is due to β-N-acetylglucosaminyltransferase 3, product of the gene *MGAT3*. The addition of GlcNAc residues either with a β1,4- or a β1,6-linkage to the two external Man residues of the core is mediated by β-N-acetylglucosaminyltransferases 4 and -5, product of the genes *MGAT4* and *MGAT5*, respectively (Figure 1A). The epigenetically regulated alternative addition of the bisecting GlcNAc, or of the β1,6-linked GlcNAc, to the N-glycans of E-cadherin [22] provides a very good example of the modulation of a basic cancer property by glycans. When bisecting GlcNAc is replaced with β1,6-linked GlcNAc on the N-glycans of E-cadherin because of decreased *MGAT3* expression β-catenin (which is usually leashed by binding to the cytoplasmic side of E-cadherin), it is released in the cytoplasm, leading to epithelial to mesenchymal transition (EMT) [23,24,25]. The methylation of the *MGAT3* promoter is responsible for *MGAT3* down-regulation and the consequent EMT [25]. In general, the presence of bisecting GlcNAc is associated with reduced malignancy, mainly because it prevents the addition of the metastasis-associated β1,6-branching. However, in some cases, an association with increased malignancy has also been reported [26]. Among epigenetic mechanisms, promoter methylation appears to play a pivotal role in inhibiting *MGAT*3 gene expression. Bisecting GlcNAc is up-regulated in ovarian cancers, while treatment with 5-AZA of ovarian cancer cell lines induces *MGAT3*, bisecting GlcNAc expression [27], and changes in the pattern of glycosylation of secreted glycoproteins [28]. Among ovarian and breast basal-like cancer patients, high *MGAT3* promoter methylation correlates with longer survival [26]. 5-AZA treatment of epatocarcinoma HepG2 cells resulted in modulation of about 20% of the glycogenes, although the consequent glycomic changes where mainly consistent with increased expression of *MGAT3* [29].

The link between β1,6 branching and metastasis is supported by a large body of evidence [30], but the underlying mechanisms are complex and only partially understood. A key role is certainly played by the preferential binding of galectin-3 to terminal structures carried by β1,6-linked branches [31]. It is not clear whether *MGAT5* is directly regulated by promoter methylation. In fact, changes in glycans of glycoproteins secreted by 5-AZA-treated ovarian cancer cells have been attributed to changes in *MGAT5* expression [28]. On the other hand, in macrophage-melanoma fusion hybrids, treatment with 5-AZA does not result in the activation of *MGAT5* transcription, but results in its down-regulation. This was explained with the demethylation-induced up-regulation of the negative regulator nm23 encoded by the *NME1* gene [32].

The addition of β1,4-linked GlcNAc, catalyzed by MGAT4, can be inhibited in mammary epithelial cells by miR-424, leading to cell cycle arrest through CCND1 down-regulation [33].

### 2.2. Mucin-Type O-Glycosylation

The addition of the first GalNAc O-linked to serine or threonine of mucin-type glycans is mediated by a family of 20 peptides: GalNAc transferases (Figure 1B), which are the products of genes *GALNT1-GALNT20*. Members of the *GALNT* family are aberrantly expressed in various cancer types, mainly as a consequence of deregulated miRNA expression.

*GALNT1* is negatively regulated by miR-129 in bladder cancer [34]. In hepatocellular carcinoma [35] and in cervical cancer cells [36], down-regulation of *GALNT4* by miR-9 promotes invasive growth. Depending on the cancer type, *GALNT7* displays opposite tumor-promoting or tumor-suppressing activities, and is regulated by different miRNAs. In laryngeal squamous cell carcinoma, it is down-regulated by miR-34a and miR-34c, with consequent reduced cell proliferation and invasion [37]. *GALNT7* promotes tumor growth also in esophageal squamous cell carcinoma, where it is overexpressed because of reduced miR-214 [38]. On the other hand, miR-30b/30d upregulation in melanoma correlates with stage, metastatic potential, shorter time to recurrence, and reduced overall survival, because of reduced expression of *GALNT7*. This leads to increased synthesis of the immunosuppressive cytokine IL-10, and to reduced immune cell activation [39]. *GALNT7* displays a tumor-suppressor effect also in hepatocellular carcinoma cells. In fact, targeting *GALNT7* by the passenger miR-17-3p enhances proliferation and migration [40]. Interestingly, the mature miR-17-5p also enhances tumor growth by targeting the tumor suppressor PTEN [40]. Decreased expression of miR-122, which is under the control of hepatocyte nuclear factor 4α in hepatitis B virus hepatocarcinoma, leads to overexpression of *GALNT10* and consequently to increased O-glycosylation and signaling through the EGF pathway [41]. In mammary epithelial cells, miR-424 down-regulates *GALNT13* [33]. miR-125a regulates ovarian cancer proliferation and invasion by repressing *GALNT14* [42].

Without further sugar additions, the O-linked GalNAc forms the Tn antigen, which is a well-known cancer-associated structure. The second step of the biosynthesis of the O-linked chains is often represented by the addition of a β1,3-linked galactose residue to GalNAc, forming the Core 1 or Thomsen-Friedenreich (or T) antigen (Figure 1B), mediated by core 1 β1,3-galactosyltransferase (C1GALT1 or T-synthase). This enzyme requires the molecular chaperone Cosmc for its activity. In the presence of a dysfunctional Cosmc, the Tn antigen accumulates. *Cosmc* expression is down-regulated by promoter methylation [43], and together with T-synthase, provides a good example of coordinated, but differential, expression. In fact, both genes are regulated by transcription factors of the same SP/Kruppel-like transcription factors, but in a B cell line only the *Cosmc* promoter undergoes methylation, despite the presence of CpG islands in the promoter region of both genes [44].

The addition of α2,6-linked sialic acid to the Tn antigen, mainly mediated by ST6GALNAC1, leads to the formation of the cancer-associated sialyl-Tn antigen and blocks further chain elongation (Figure 1B). The *ST6GALNAC1* gene undergoes hypermethylation out of the promoter region in breast cancer patients positive for both estrogen and progesterone receptors, but not in those negative [45]. The effect of such differential DNA methylation on ST6GALNAC1 expression is not reported yet. Conversely, *ST6GALNAC1* down-regulation was reported in esophageal squamous cell carcinoma patients, and was found to be associated with promoter methylation in cell lines [46].

### 2.3. Gangliosides

Gangliosides are sialylated glycolipids whose expression is frequently deranged in cancer [47]. The final sialylation of gangliosides is mediated by several sialyltransferases, some of which are regulated by promoter methylation or miRNAs. In prostate cancer cell lines, the biosynthesis of ganglioside GD1a (Figure 1C) is regulated by sialyltransferase ST3GAL2, whose promoter methylation blocks the transcriptional stimulation operated by testosterone [48]. Sialyltransferase ST6GALNAC5, responsible for GD1α biosynthesis, displays CpG hypermethylation in both adenomas and carcinomas of the colon, but without effect on gene expression [49]. In one study, it was shown that three glycosyltransferases, ST3GAL5, ST6GALNAC5, and B3GLCT (the first two involved in ganglioside biosynthesis, the third in elongation of O-linked fucosylglycans), affect EMT, and are regulated by miRNAs of the miR-200 family. Silencing of these glycogenes induces EMT, like the expression of miR-200f, indicating a causal role of these gangliosides in EMT [50]. A very recent study confirmed that targeting the 3′UTR of ST3GAL5 by miR-26a, miR-548l and miR-34a downregulates enzyme expression and the growth of hepatocarcinoma cells [51].

### 2.4. Type 1 and Type 2 Chain Elongation

**Figure 2:**
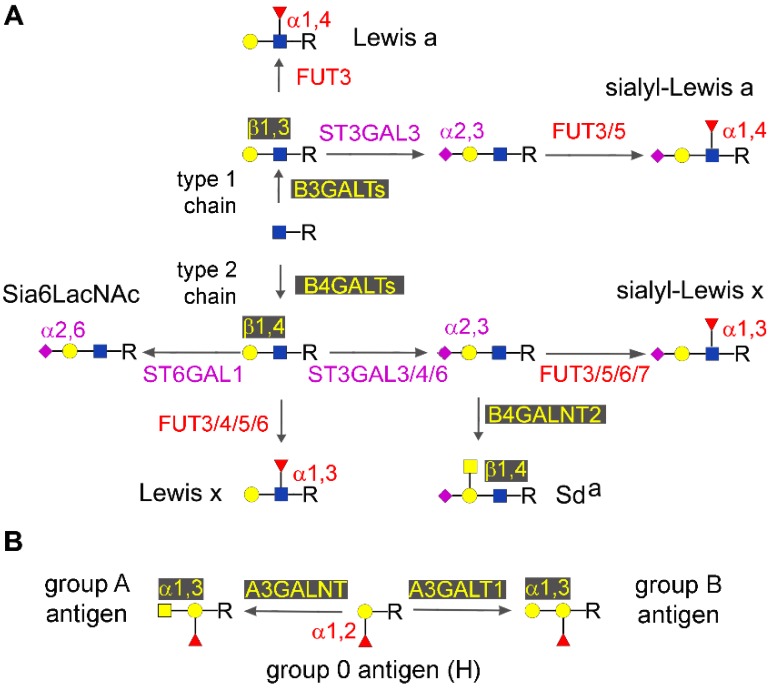
Structure of some oligosaccharide chain terminations. Monosaccharides are depicted as in Figure 1. Anomers, linkage positions, and enzymes involved in relevant reactions are indicated. (**A**) Origin of type 1 and 2 chains, and termination by bioactive ends; (**B**) AB0 antigens.

Both N- and O-glycans can be elongated by repeating units of galactose linked to GlcNAc either through a β1,3 or a β1,4 linkage, giving rise to type 1 or type 2 (lactosamininc) chains, respectively (Figure 2A). Several β1,3-galactosyltransferases (B3GALTs) or β1,4-galactosyltransferases (B4GALTs) mediate the elongation of type 1 or type 2 chains, respectively, and are regulated by epigenetic mechanisms. B3GALT5, which is responsible for switching oligosaccharide elongation to the type 1 chain in many epithelial cells, provides quite a complex example of epigenetic regulation. Two main transcripts were described with different 3′ UTR, but with an identical coding sequence. One, named the native transcript since it is conserved through the evolution, is driven by a promoter affected by the ubiquitous NF-Y transcription factor, and is placed in the context of two CpG islands [52]. In colon cancer, hypermethylation of the native promoter results in gene silencing [53]. The same promoter appears to be hypomethylated in both normal and cancer pancreatic tissues, allowing relevant expression of the transcript [54]. The other *B3GALT5* transcript, named the LTR transcript due to its retroviral origin, appeared late in the evolution [55], and is driven by a promoter affected by HNF1 transcription factor [56]. This promoter is very strong in normal colon mucosa giving rise to a very high expression of *B3GALT5*, but is silenced in colon cancer due the demethylation of a distant DNA sequence [56], probably located about 1 kb far from the transcription initiation site [54]. Treating cell lines that express the LTR transcript with 5-AZA dramatically impairs *B3GALT5* expression. In the pancreas, the LTR transcript is expressed at low levels and is not apparently regulated in cancer, where the same DNA region is not differentially methylated [54]. Among other galactosyltransferases regulated in cancer by epigenetic mechanisms, *B4GALT1* is down-regulated because of promoter methylation in a significant percentage of colon cancer cases [57]. An unconventional example of stimulation of gene expression by miRNA is provided by miR-27a, which binds to the 3’UTR of B4GALT3, resulting in its overexpression, and contributing to its oncogenic activity in cervical cancer cells [58].

The *B3GNT7* gene encodes β1,3-N-acetylglucosaminyltransferase 7, that mainly acts on extending sulfated polylactosaminic chains (Figure 1A) on which sLe^x^ and sLe^a^ antigens are mounted. The down-regulation of B3GNT7 observed in colon cancer is partially due to promoter methylation [59].

### 2.5. Type 1 and type 2 Chain Termination

#### 2.5.1. Major Glycosyltransferases Involved in Chain Termination

The termination of type 1 or type 2 chains frequently occurs by the addition of a few types of monosaccharides, arranged in well-defined structures recognized by antibodies or lectins. The addition of these monosaccharides is mediated by specific glycosyltransferases, many of which display epigenetic regulation. Sialic acids are a group of sugars carrying a negative electric charge at physiological pH values always mounted in terminal position, except in the case of polysialic acids, where sialic acid residues form linear polymers. Sialyltransferases, the enzymes which transfer sialic acids, are a family of 20 enzymes which can be grouped according to the sugar acceptor (Gal, GalNAc, GlcNAc, Sia) and the position (α2,3-α2,6-α2,8-) of the linkage they form [60,61,62].

Members of the fucosyltransferase family [63] mostly involved in the elaboration of terminal epitopes are FUT1 and FUT2, which mediate the α1,2-fucosylation of terminal galactose, and FUT3-10, which catalyze the α1,3/4-fucosylation of the subterminal GlcNAc residue, giving rise to the group of Lewis antigens [63,64,65].

Relevant examples of chain termination are provided by the α2,3-sialylated/α1,3/4 fucosylated sialyl-Lewis type antigens, the Sd^a^ antigen, the Sia6LacNAc, and the well-known AB0 antigens.

#### 2.5.2. Lewis and Sd^a^ Antigens

The biological relevance of Lewis type, and in particular of sialyl-Lewis (sLe) type antigens, in cancer biology is vast [64] and is mainly related to the ability of these structures to serve as ligands for cell adhesion molecules of the selectin family, favoring the metastatic process and angiogenesis [66]. The terminal steps of the biosynthesis of sLe structures are represented by the α2,3-sialylation of type 1 or type 2 chains, and by the successive addition of fucose to GlcNAc in α1,4- or α1,3 linkage, yielding sLe^a^ and sLe^x^, respectively. Both the sialylation and fucosylation steps can be mediated by multiple enzymes (Figure 2A).

Among the reasons for the low expression of the sLe^a^ antigen in normal colonic tissues, an alternative biosynthesis of the disialyl-Lewis structure has been proposed, in which an additional α2,6-linked sialic acid is mounted on the GlcNAc residue by the action of sialyltransferase ST6GALNAC6 [67]. Treatment of the colon cancer cell line DLD-1 with 5-AZA and/or butyrate up-regulates the transcript, suggesting that promoter methylation and/or histone deacetylation are involved in its regulation [67]. In a gastric cancer cell line, Le^a^ is regulated by the levels of FUT3 that appear to be reduced by methylation of the promoter region [68]. The differential methylation of the *FUT3* promoter was recently reported to occur in tongue cancer, and to be associated with some clinical features [69]. *ST3GAL3*, coding a sialyltransferase involved in the biosynthesis of type 1 chain active ends including sLe^a^ in several tissues, was recently reported to be differentially methylated during childhood [70], but data specifically related to cancer are not yet available. In a colon cancer cell line, 5-AZA treatment induced sLe^x^ expression on MUC1 by stimulating ST3GAL6 transcription, which is impaired by promoter methylation [71]. Among the mechanisms claimed to be responsible for low sLe^x^ expression in normal colon, there is the biosynthesis of two alternative structures. One is sialyl 6-sulfo sLe^x^, which critically depends on the activity of the sulfate transporter DTDST, whose down-regulated in colon cancer tissues is restored by histone deacetylase inhibitors [72].

The other structure is the Sd^a^ antigen (Figure 2A), which is formed by a α2,3-sialylated type 1, 2 or 3 chain in which a GalNAc residue is β1,4-linked to subterminal galactose (Figure 2A) [73]. The terminal step of the biosynthesis of the Sd^a^ antigen, is mediated by a single enzyme, a product of the *B4GALNT2* locus [74,75]. This enzyme and its cognate Sd^a^ antigen mediate multiple biological and pathological functions [76]. The putative promoter regions of the human *B4GALNT2* gene are embedded in CpG islands, suggesting that DNA methylation could play a role in regulating *B4GALNT2* gene expression, particularly in the down-regulation observed in gastrointestinal cancers [77,78,79]. It has been reported that in colon cancer cells lines, *B4GALNT2* promoter methylation is associated with a very low level of mRNA expression. Further, demethylating treatments can induce a partial activation of the gene, reaching an expression level which remains far below that of the normal colonic mucosa [80]. The *B4GALNT2* gene was found to be methylated in about half of the gastric cancer cases examined, and in the majority of gastric and colon cancer cell lines [81]. Treatment of cell lines with anti DNA-methylation agents induced a weak expression of the *B4GALNT2* transcript, and of the Sd^a^ antigen [81]. Altogether, these results are consistent with the view that methylation of the *B4GALNT2* genomic regulatory region plays a role in switching off gene expression during carcinogenesis of gastrointestinal tissues, but that the removal of epigenetic marks is not sufficient to drive the level of enzyme activation close to that of normal colonic mucosa. Taken together, the down regulation of sLe^a^ [82] and Sd^a^ antigens, the up regulation of sLe^x^ [83] in colon cancer, and the persistent expression of some Lewis antigens in pancreatic cancer [54], appear as the consequence of a complex tissue- and cancer-specific regulation of several glycogenes operated by various epigenetic mechanisms. At present, they cannot be recapitulated in a simplified minimal cancer-associated signature. Consequently, a single oriented approach, i.e general DNA demethylation obtained through methyltransferase inhibitors, could be not useful in these types of cancers.

Of the fucosyltransferases involved in the biosynthesis of Lewis type antigens, the methylation of *FUT4* promoter has been reported to be inversely correlated with gene overexpression and tumor invasion [84]. In breast cancer cells, FUT4 overexpression, due to down-regulation of miR-224-3p, is associated with chemoresistance [85], while its inhibition by miR-493-5p attenuates invasiveness and tumorigenicity [86]. Although FUT6 is involved in the biosynthesis of the cancer associated sLe^x^ antigen, it has also been reported that FUT6 down-regulation induced by miR-106b targeting leads to increased invasion of breast cancer cells [87].

#### 2.5.3. Sia6LAcNAc and ST6GAL1

The termination of type 2 chains with α2,6-linked sialic acid (Figure 2A), giving rise to α2,6 sialyllactosamine (Sia6LacNAc), is mainly mediated by sialyltransferase *ST6GAL1* [88]. Sia6LacNAc decorates a variety of soluble and cell membrane glycoproteins, and plays roles in cell adhesion [89] and immune and inflammatory processes [90,91,92] (reviewed in [93]).

Sialyltransferase *ST6GAL1* is frequently up-regulated in colon cancer [94] and other malignancies, but its impact on cancer biology is multifaceted [60,61,93,95,96]. *ST6GAL1* down-regulation by promoter methylation has been documented in bladder cancer [97], in breast cancer patients ER/PR positive, with TP53 mutations and high grade [98], and in gliomas [99]. In all these cases, *ST6GAL1* down-regulation is associated with increased invasion.

An example of how miRNA can indirectly modulate a biological function by targeting glycosylation is provided by miR-199a, which reduced both the sialylation and the protein level of Nectin-like Molecule 2/Cell Adhesion Molecule 1 by targeting *ST6GAL1* [100].

#### 2.5.4. Other Sialyltransferases

In breast cancer cells, the overexpression of sialyltransferase ST8SIA4, involved in polysialic acid biosynthesis, is associated with invasive growth. A main regulatory mechanism involves miR26a-26b, which targets the 3′UTR region of the transcript determining its down-regulation [101]. Interestingly, ST3GAL6, whose upregulation in hepatocarcinoma is associated with increased invasion, is also targeted and negatively regulated by miR26a [102]. It has been reported that miR-4701-5p is responsible for multi drug resistance of chronic myeloid leukemia cells, at least in part, by targeting and down-regulating ST3GAL1 [103]. The invasive properties and tumorigenicity of human follicular thyroid carcinoma is mediated by miR-4299, through targeting and silencing ST6GALNAC4 [104].

#### 2.5.5. AB0

Histo-blood group AB0 antigens (Figure 2B) derive from either the addition of α1,3GalNAc residue (group A), an α1,3Gal residue (group B), or nothing (group 0), to an α1,2-fucosylated terminal galactose (Figure 2B). Three allelic variants of the *ABO* genetic locus encoding either an α1,3GalNAc transferase (*A3GALNT*), an α1,3Gal transferase (A3GALT), or an inactive molecule, are responsible for the A, B and 0 phenotypes, respectively [105,106].

The expression of AB0 antigens in bladder cancer is frequently lost either because of allelic lost or because promoter methylation of the AB0 gene [107]. The fact that the AB0 gene is silenced in about two-thirds of the oral cancer cases [108] suggests that its down-regulation provides a growth advantage [109]. However, in only one-third of the cases, silencing is due to promoter hypermethylation, being the remaining due to allelic lost or other mechanisms [108]. AB0 promoter hypermethylation was also found in hyperplastic or dysplastic tissues adjacent to the tumors, suggesting that it is an early event in tumorigenesis [108]. Downregulation of microRNA-15b by hepatitis B virus X enhances hepatocellular carcinoma proliferation, via FUT2-induced Globo H expression [110].

### 2.6. Glycosaminoglycans

Glycosaminoglycans are important regulators of the growth of connective tissue cells. In chondrosarcoma, heparan sulfate biosynthesis is inhibited because of the methylation of the 3-O-sulfotransferase [111]. The exostoses like-3 *(EXTL3*) gene encodes a putative tumor suppressor glycosyltransferase involved in the biosynthesis of glycosaminoglycans. In a high percentage of the colon cancers of the mucinous type, *EXTL3* is down-regulated by promoter methylation. This results in the loss of heparan sulfate with a probable impact on the mechanisms of growth control [112]. On the contrary, hypermethylation of *EXTL3* promoter has never been reported in non-mucinous colon cancers.

## 3. Epigenetic Regulation of Sugar binding Molecules: The Galectins

Galectins are a family of 15 galactose binding proteins that regulate many aspects of the cell life through sugar-dependent, but also sugar independent, interactions. The different members of the galectin family can play opposite effects by promoting cell growth or inducing apoptosis. The role of galectins in cancer and immune regulation is well-known [113].

### 3.1. Galectin 1

The gene *LGALS1*, encoding galectin-1, is usually hypermethylated in colorectal cancer cells—its induction by demethylating treatments induces apoptosis because of down-regulation of Wnt signaling [114]. Hepatic stellate cells play a role in hepatocarcinoma development by locally suppressing the immune response. In hepatic stellate cells of hepatocarcinoma patients, galectin-1 is much higher than in those from normal liver. Meanwhile, in MiR-22, which targets galectin-1, was lower [115], indicating its role in the regulation of the anti-cancer immune response.

### 3.2. Galectin 3

The *LGALS3* gene, encoding galectin-3, shows promoter methylation-dependent regulation in different cancers. It is unmethylated in normal and benign prostate tumors, but it is silenced by methylation in prostate cancer [116], albeit in a stage-specific manner. In fact, it is heavily methylated in stage I and II adenocarcinoma, and lightly methylated in stage III and IV, allowing discrimination of the stages [117]. Promoter methylation is a major mechanism of *LGALS3* gene silencing also in mucinous colorectal cancers [118], as well as in pituitary tumors, and breast and thyroid cancer cell lines [119]. Conversely, the average hypomethylation of five CpG sites in the promoter sequence is associated with high *LGALS3* expression in thyroid cancer, and allows to distinguish it from normal thyroid tissues [120]. One of the reasons for galectin-3 overexpression in colorectal cancer, which is associated with disease progression and shorter survival, is the decreased levels of the inhibitory miR-128 [121]. Mucin MUC-1 and Galectin-3 provide an example of a self-fueling loop involving a miRNA. MUC1 is transmembrane glycoprotein whose Asn36 residue of the C-terminal subunit can be decorated by an N-linked chain. Owing to its dimeric nature, galectin-3 can bound in both Asn35 of MUC1 and EGFR, activating a signal transduction pathway leading to the suppression of miR-322. Down-regulation of miR-322 stabilizes galectin-3 transcript, leading to increased galectin-3 levels with a positive feedback on MUC1 signaling [122].

### 3.3. Galectin 7

A few reports document regulation of the *LGALS7* gene, encoding galectin-7, in cancer. Promoter hypomethylation is at the basis of the up-regulation of galectin-7 during lymphoma progression [123], while promoter hypermethylation is responsible for galectin-7 downregulation in gastric cancer, a tumor in which it has a tumor suppressive function [124].

### 3.4. Galectin 9

Galectin-9 has been reported to exert anticancer activity in gallbladder cancer by modulation of the miRNA expression profile [125]. On the other hand, in liver cancer, galectin-9 is overexpressed because of miR-22 downregulation. As a consequence, lymphocyte apoptosis is increased and tumor growth is promoted [126].

## 4. Glycosylation as a Part of the Epigenetic Code: O-GlcNAc

In the previous sections, we reviewed how epigenetic mechanisms can regulate the expression of a variety of glycogenes. In this last section, we describe how a very peculiar type of glycosylation, the O-linked GlcNAc (O-GlcNAc), is itself part of the epigenetic code. The addition of O-GlcNAc is basically different from the “conventional“ N- and O-glycosylation for the following reasons. GlcNAc is added to cytoplasmic and nuclear (rather than membrane or secreted) proteins by the action of a single enzyme: O-GlcNAc transferase (OGT), and is removed by the action of O-GlcNAc ase (OGA). The addition/removal of O-GlcNAc is a dynamic and reversible process which prevents phosphorylation of the Ser/Thr residues, to which O-GlcNAc is attached. The OGT activity is crucially dependent on the availability of its donor substrate UDP-GlcNAc, whose concentration is directly linked to the nutritional state of the cell. O-GlcNAc can be added to transcription factors involved in embryonic development [127] and histones [128], with a large impact on gene expression. A very recent paper reports that the inhibition of OGT results in a reduction of colon cancer stem cells population. At least in part, this phenomenon is mediated through the O-GlcNAc-mediated methylation of the CpG islands in the promoter region of the *MYBL1* gene, encoding a transcription factor which inhibits colon cancer stem cells growth [129]. On the other hand, OGT itself is epigenetically regulated, being a target of miR-424 [33].

## 5. Concluding Remarks

**Figure 3:**
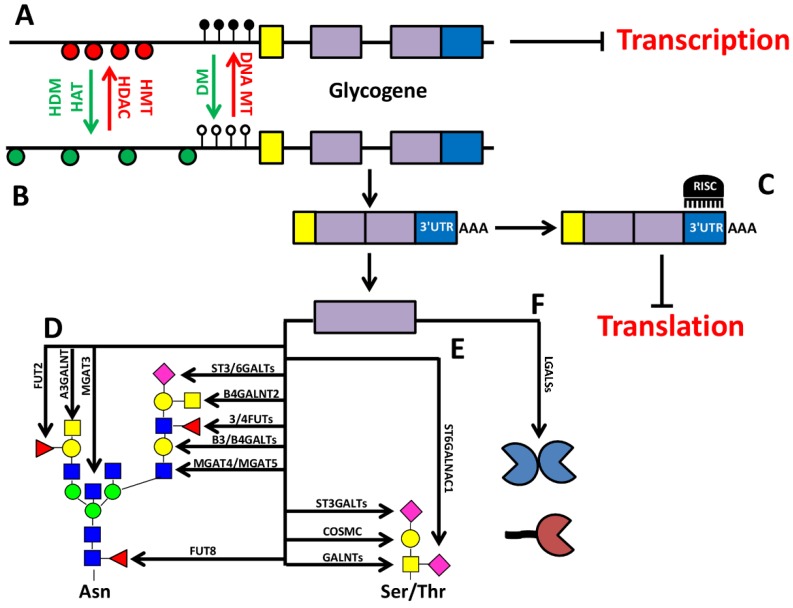
Summary of the common epigenetic mechanisms of glycosylation control. A hypothetical glycogene is represented in which the activity of histone methyltransferase (HMT), histone deacetylase (HDAC), and DNA methyltransferase (DNA MT) results in chromatin condensation determining a transcriptionally inactive state; closely spaced red circles represent nucleosomes, black lollipops represent methylated cytosine residues (**A**). The gene can be turned into a transcriptionally active state by the activity of histone demethylase (HDM) and histone acetyltransferase (HAT), which reduce chromatin condensation (loosely spaced green circles), and by demethylation of the promoter region (white lollipops) (**B**). Transcription of the gene results in a mRNA comprised of a 5′UTR (yellow), a protein coding region (violet), and a 3′UTR (blue), which can be targeted by miRNA, resulting in translation inhibition; RISC, RNA-induced silencing complex (**C**). This hypothetical glycogene is a paradigm of many others controlled by epigenetic mechanisms. They are involved in a variety of biosynthetic steps that are grouped in the hypothetic N- and O-linked structures depicted in (**D)** and (**E**). The epitopes shown in (**D**) and (**E**) are often mutually exclusive, and are presented here as a single structure only for didactic purposes. Moreover, glycogenes encode molecules like galectins (**F**) involved in the biological roles of glycans.

The review of recently published data showed that many cancer-associated alterations of the glycan profile of cells and tissues depend on the epigenetic deregulation of glycogenes. They include the methylation of promoter sequence, and the aberrant expression of miRNAs, as the most frequent mechanisms, both acting in many cases as inhibitors of glycogene expression (Figure 3). As originally shown for tumor suppressor genes, in cancer there is a general hypermethylation of the promoter of many genes, determining their transcriptional inhibition, and favoring the malignant phenotype.

Gene reactivation can be obtained by DNA demethylating agents, restoring the normal levels of gene expression. However, relevant exceptions exist, and can be useful to shed light on the general mechanisms of epigenetic control. In fact, promoter hypermethylation can be tissue specific and evident in cancer cell lines, but not in the corresponding native tumors [46]. Treatment with demethylating agents, which is usually the first approach in determining whether a gene is subjected to promoter methylation, sometimes appear unable to counteract hypermethylation and/or to restore transcription levels [49,56,116,120]. Moreover, treatment with demethylating agents inhibits, instead of stimulating, several glycogenes [28,54,99]. The opposite role of methylation of specific CpG pairs in promoters, or in distinct DNA regions far from the promoter sequences, should be kept in mind in this regard [45,56,120]. Furthermore, some of the glycogenes reactivated through demethylating agents, and some of those inhibited by the same treatment do not attenuate, but instead sustain the malignant phenotype [26,32,71], an observation relevant in view of the clinical use of such agents. The complexity of the picture is recapitulated by the expanding role of miRNAs that also appear to be able to determine double-edged effects on glycogenes (Table 1). Being more specific and straightforward, they are thus promising tools for therapies aimed at the inhibition of such genes.

## Figures and Tables

**Table 1:** List of glycogenes regulated by epigenetic mechanisms.

Target	Epigenetic Mechanism	Effect	Tissues/Cells Involved	Reference
*Galactosyltransferases*				
A3GALT1/A3GALNT	Promoter methylation	Down-regulation	Bladder and oral cancer	[107,108]
B3GALT5 native	Promoter methylation	Down-regulation	Colon cancer	[53,54]
B3GALT5 LTR	Distant sequence methylation	Up-regulation	Colon cancer	[54,56]
B4GALT1	Promoter methylation	Down-regulation	Colon cancer	[57]
B4GALT3	miR-27a	Up-regulation	Cervical cancer	[58]
C1GALT1 chaperone Cosmc	Promoter methylation	Down-regulation	B lymphocyte and other model cell lines	[43,44]
*N-acetyl-galactosaminyl transferases*				
GALNT1	miR-129	Down-regulation	Bladder cancer	[34]
GALNT4	miR-9	Down-regulation	Liver and cervical cancer	[35,36]
GALNT7	miR-34° -34c	Down-regulation	Laryngeal cancer	[37]
	miR-214	Down-regulation	Esophageal cancer	[38]
	miR-17-3p	Down-regulation	Liver cell lines and mouse model	[40]
GALNT10	miR-122	Down-regulation	Liver cancer	[41]
GALNT13	miR-424	Down-regulation	Breast and HEK293 cell lines	[33]
GALNT14	miR-125a	Down-regulation	Ovarian cancer	[42]
A3GALNT/A3GALT1	Promoter methylation	Down-regulation	Bladder and oral cancer	[107,108]
B4GALNT2	Promoter methylation	Down-regulation	Gastrointestinal cancer	[80,81]
*N-acetyl-glucosaminyl transferases*				
MGAT3	Promoter methylation	Down-regulation	Mammary model and ovarian and liver cell lines	[25,26,27,28,29]
MGAT4	miR-424	Down-regulation	Breast and HEK293 cell lines	[33]
MGAT5	Distant methylation	Down-regulation	Ovarian cell line	[28]
MGAT5	Distant methylation	Up-regulation	Melanoma hybrid cell line	[32]
B3GLCT	miR-200	Down-regulation	Breast cell line	[50]
B3GNT7	Promoter methylation	Down-regulation	Colon cancer	[59]
OGT	miR-424	unclear	Breast and HEK293 cell lines	[33]
*Sialyltransferases*				
ST3GAL1	miR-4701-5p	Down-regulation	Chronic myeloid leukemia	[103]
ST3GAL2	Promoter methylation	Down-regulation	Prostate cell lines	[48]
ST3GAL3	Promoter methylation	unknown	Whole methylome	[70]
ST3GAL5	miR-200	Down-regulation	Breast cell line	[50]
	miR-26a, miR-548l, miR-34a	Down-regulation	Liver cancer	[51]
ST3GAL6	Promoter methylation	Down-regulation	Colon cancer cell line	[71]
	miR-26a	Down-regulation	Liver cancer	[102]
ST6GAL1	Promoter methylation	Down-regulation	Glioma, bladder and breast cancer	[97,98,99]
	miR-199a	Down-regulation	Lung and HEK293 cell lines	[100]
ST6GALNAC1	Promoter methylation	Down-regulation	Esophageal cancer	[46]
ST6GALNAC4	miR-4299	Down-regulation	Thyroid cancer	[104]
ST6GALNAC5	Promoter methylation	No regulation	Colon cancer	[49]
	miR-200	Down-regulation	Breast cell line	[50]
ST6GALNAC6	Putative promoter methylation/ histone deacetylation	Down-regulation	Colon cancer	[67]
ST8SIA4	miR26a-26b	Down-regulation	Breast cancer	[101]
*Fucosyltransferases*				
FUT2	miR-15b	Down-regulation	Liver cancer	[110]
FUT3	Promoter methylation	Down-regulation	Gastric and tongue cell lines	[68,69]
FUT4	Promoter methylation	Down-regulation	Skin cell lines	[84]
	miR-224-3p	Down-regulation	Breast cancer	[85]
	miR-493-5p	Down-regulation	Breast cancer	[86]
FUT6	miR-106b	Down-regulation	Breast cancer	[87]
FUT8	miR-198	Up-regulation	Colon cancer	[19]
	miR-122 -34a 26a -146a	Down-regulation	Liver cancer	[20,21]
*Sulfotransferases*				
HS3STs	Promoter methylation	Down-regulation	Chondrosarcoma cell line	[111]
*Nucleotide donor transporters*				
DTDST	Histone deacetylation	Down-regulation	Colon cancer	[72]
*Galectins*				
LGALS3	Promoter methylation	Down-regulation	Colon, prostate and pituitary cancer	[16,17,18,19,20,21,22,23,24,25,26,27,28,29,30,31,32,33,34,35,36,37,38,39,40,41,42,43,44,45,46,47,48,49,50,51,52,53,54,55,56,57,58,59,60,61,62,63,64,65,66,67,68,69,70,71,72,73,74,75,76,77,78,79,80,81,82,83,84,85,86,87,88,89,90,91,92,93,94,95,96,97,98,99,100,101,102,103,104,105,106,107,108,109,110,111,112,113,114,115,116,117,118,119]
	Promoter hypomethylation	Up-regulation	Thyroid cancer	[120]
	miR-322	Down-regulation	Breast, lung, prostate and HEK293 cell lines	[122]
	miR-128	Down-regulation	Colon cancer	[121]
LGALS7	Promoter methylation	Down-regulation	Gastric cancer and lymphomas	[123,124]
LGALS9	miR-22	Down-regulation	Liver cancer	[126]

## Abbreviations

5-AZA	5-Aza-2′-deoxycytidine
EMT	Epithelial to mesenchymal transition
